# Power Slap Competitions as a Novel Mechanism of Traumatic Injury

**DOI:** 10.7759/cureus.93842

**Published:** 2025-10-04

**Authors:** Cayla Guerra, Chase Edison, Samuel Jang, Joshua J Oliver

**Affiliations:** 1 Department of Emergency Medicine, Madigan Army Medical Center, Tacoma, USA

**Keywords:** contact sport, mandibular fractures, maxillofacial trauma, novel traumatic mechanism, power slap, sports lover, sports-related injuries, trauma, ufc, ultimate fighting championship

## Abstract

Professional Power Slap fighting has been an official Ultimate Fighting Championship (UFC) sport since January of 2023. Competitors both deliver and sustain repeated open-hand slaps to the face, often resulting in knockouts (KOs). The sport is growing in popularity, but there is currently no medical literature describing Power Slap fighting and the unique traumatic injuries it can precipitate. Power Slap fighting is a novel mechanism of injury, which has a different set of considerations than other UFC sports. This is the first documented case of a patient presenting after an unofficial and unsanctioned bout of Power Slap fighting and the traumatic craniofacial sequelae that befell him. He presented to the emergency department (ED) in significant pain with trismus and dental trauma requiring urgent surgical management. His course was complicated by required revision surgery and likely permanent nerve damage. His case and the lessons learned by those participating in his care are useful to future ED clinicians caring for their own Power Slap patients.

## Introduction

For as long as humans retain a corporeal form, they will continue to discover new ways to sustain traumatic injuries. Power Slap fighting is one such mechanism, a sanctioned sport in which participants deliver open-handed blows to the face. Ultimate Fighting Championship (UFC) officially adopted Power Slap fighting in January of 2023 [1]. Despite rapid growth in popularity, its injury patterns remain undocumented. Matches consist of two competitors alternating roles as the striker and defender. The striker delivers a measured slap to the target zone (between the chin and eye line) while the defender must remain still and unguarded. Fouls may occur for striking outside this zone ('clubbing') or for protective movements by the defender ('flinching'). Safety protocols require minimal protective gear, including mouthguards and cotton ear inserts, and licensed participants must undergo a screening process, including brain imaging, baseline labs, and ophthalmologic exam [1]. Combat sports such as mixed martial arts (MMA) and the UFC have been associated with high rates of traumatic brain injury (TBI), facial fractures, and dental trauma [2-5]. However, Power Slap differs substantially in its mechanics: all force is directed to the unprotected lower face, and the defender cannot evade or defend themselves. Here, we present the first known case of a patient sustaining significant facial trauma during an unsanctioned Power Slap event. This case is furthermore unique as he is a United States Army service member. His course included surgical repair, revision, and long-term neurologic sequelae. This case highlights the need for clinical awareness of Power Slap fighting as a unique mechanism of injury.

## Case presentation

**Figure 1:**
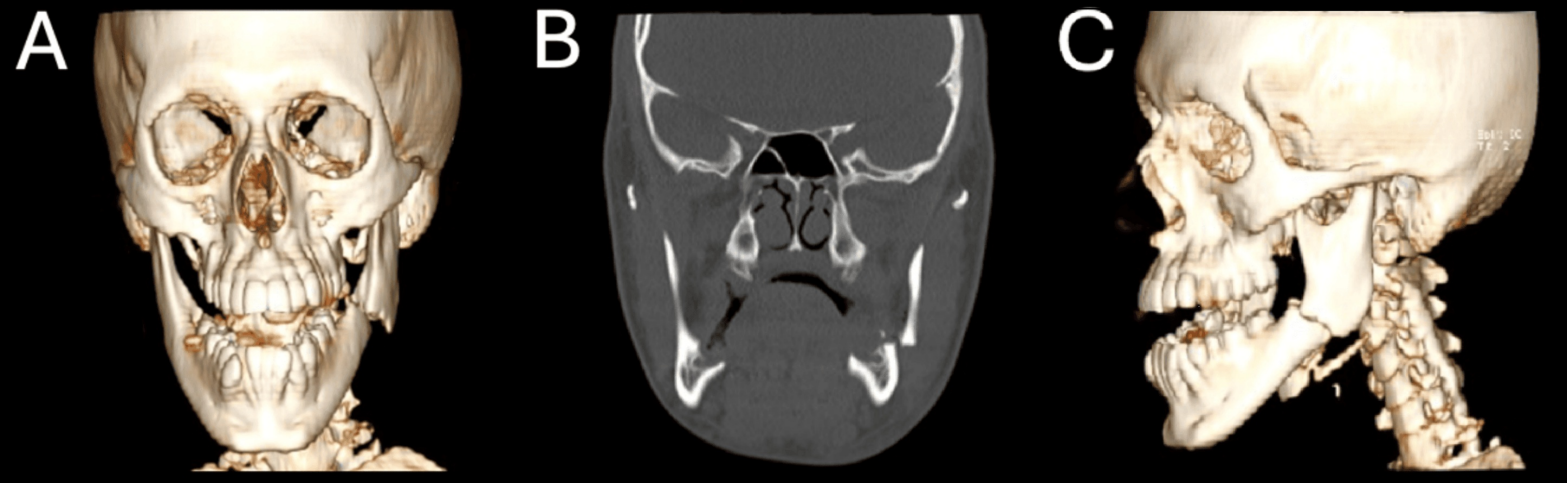
Acute left mandibular fractures on imaging with 3D reconstruction (A) CT maxillofacial with 3D reconstruction (coronal view) showing a mildly comminuted fracture of the angle of the left mandible. (B) CT maxillofacial (coronal view) demonstrating an acute, mildly comminuted fracture of the left mandibular angle, along with an old right mandibular fracture at the anterior body adjacent to the mental foramen. (C) CT maxillofacial with 3D reconstruction (lateral view). CT: computed tomography; 3D: three-dimensional

This was an otherwise healthy 20-year-old male patient brought to the emergency department (ED) with left jaw pain, facial swelling, and trismus. He reported that he had been involved in an amateur Power Slap fighting competition prior to presenting. His airway was intact, he was breathing spontaneously, and had no major bleeding. He was alert, oriented, but only able to communicate by head shaking and hand gestures. He denied loss of consciousness, ear pain or tinnitus, vision changes, paresthesia, and neck pain. He had reassuring vital signs; initially, his blood pressure was 121/60 mmHg, heart rate was 49 beats per minute, respiratory rate was 18 breaths per minute, and oxygen saturation was 97% on room air, with a temperature of 36.5 degrees Celsius. Physical exam demonstrated exquisite tenderness to palpation along the left mandible, moderate trismus, and subluxation of several teeth on the left mandible. There was some small blood oozing from the mouth, but the patient continued to protect his airway without issue. There was no ear canal trauma and no hemotympanum. Laboratory testing in the ED revealed hemoglobin/hematocrit within normal limits, mild leukocytosis to 12.3 × 10^3^/μL, and a comprehensive metabolic panel within normal limits. Due to concerning physical exam, a maxillofacial computed tomography (CT) was obtained. This scan demonstrated a comminuted fracture at the angle of the left mandible (Figure 1). 

With pain control, the patient's communication ability improved. He was administered 1 g of intravenous (IV) acetaminophen, 2 mg total of IV hydromorphone, 4 mg of IV ondansetron, 1 L of sodium chloride 0.9%, and 1 g of IV ceftriaxone. Surgical consultation with an otolaryngology (ENT) provider was pursued. They evaluated the patient and recommended urgent surgical intervention for the patient’s mandibular fracture. The patient consented and was taken to the operating room (OR) for open reduction and internal fixation (ORIF) of the fracture the same day. The intraoperative report described good anatomic reduction but poor occlusional reduction, which initially appeared to be similar to the patient’s baseline. During the procedure, the surgeon confirmed the presence of a prior healed right-sided mandibular fracture. The patient reported on follow-up that he had sustained multiple prior impacts to the jaw.

Postoperatively, he continued to have malocclusion. Due to this, he required revision open reduction and plating of bilateral mandibles. At his two-month follow-up, he had residual loss of sensation in the bilateral mandibular nerve distribution. The surgical team believed that the loss of sensation may eventually recover on the right, but it will likely be permanent on the left.

## Discussion

The majority of the existing literature surrounding craniofacial trauma in UFC competition centers around MMA. However, the patterns of injury and considerations for an athlete injured during a Power Slap fight differ in some important ways. TBI was the most sustained injury in UFC sports before Power Slap fighting and was significantly more frequently reported than fractures [2]. The UFC had no formal concussion protocol before 2021, which meant that the management of these high-prevalence injuries was not standardized [3]. This protocol was instituted just over one year prior to the inception of Power Slap fighting, so no long-term conclusions can be drawn regarding its effectiveness in preventing lasting effects from TBIs. Knockouts (KOs) are associated with significantly higher rates of TBI as well as fractures, and KOs are a commonly sought-after outcome in Power Slap fighting [4]. Therefore, it stands to reason that TBIs and fractures will occur frequently in Power Slap participants. Among fractures, the most common type of craniofacial injury reported is trauma to the upper and middle face [5]. However, the Power Slap competitors are striving only to slap the lower face. The first documented case of trauma from Power Slap fighting is not enough to determine the prevalence of lower facial trauma. However, it is a reminder that facial fractures should be a top consideration for patients injured during this sport. Our recommendations on the approach to patients presenting with craniofacial fractures sustained from Power Slap fighting are outlined below. 

Assessing for airway patency is the most important first step. In cases of mandibular fracture, a rare but feared consequence is acute airway compromise [6]. Therefore, accurate initial evaluation and ongoing monitoring for any signs of respiratory distress are imperative. In any case of craniofacial trauma, the airway assessment needs to include a thorough oropharyngeal examination to evaluate for injuries or missing teeth. A portable chest X-ray (CXR) may be considered to evaluate for tooth aspiration should there be significant dental trauma or teeth that cannot be accounted for [7]. Lastly, unlike most other UFC sports, Power Slap fighters have very minimal protective equipment requirements. This includes cotton ear protectors for inner ear protection. Unfortunately, cotton ear protectors are an insufficient method of barotrauma prevention and will not appropriately protect the tympanic membrane or inner ear from injury that may be sustained during a Power Slap competition, making an ear exam necessary [8].

Pain management also plays a large role in the ED, and multimodal pain management is the mainstay of treating acute traumatic musculoskeletal pain [9]. Given the risk of airway compromise in lower facial fractures, it is important to balance the safety and efficacy of pain control treatment. Using opioids will likely be necessary, but care must be taken to avoid extended-release opioid agents. Preference is given to immediate-release opioids for the shortest duration, ensuring a better ability to manage airway and breathing should the need arise. Additionally, for higher-risk patients or patients who will stay in the ED for a prolonged time prior to operative management, an appropriate nerve block can be considered [9]. In our patient’s case, adequate analgesia was achieved with 1 g of acetaminophen and 2 mg total of hydromorphone. The opioid was administered in separate doses, with careful reassessment between redosing.

Importantly, tetanus status should be ascertained, and tetanus vaccine should be updated if it has been over five years since administration [10]. However, the administration of antibiotics in the setting of a mandibular fracture has long been disputed. The definition of an ‘open’ versus ‘closed’ fracture near the oral mucosa has not been definitively outlined in the literature. Recent large meta-analyses have concluded that there is weak evidence to support the use of preoperative prophylactic antibiotics in maxillofacial fractures [11-13]. Due to this weak evidence and the broad variation in types of facial fractures, we recommend that the ED provider consider antibiotic administration. There are no formal guidelines for antibiotic selection. In this case, our patient was provided with 1 g of IV ceftriaxone. We recommend Unasyn administered for the added benefit of oral flora gram-negative coverage.

We recommend that all ED clinicians make themselves aware of their available consultants, transfer criteria, and the patient's socioeconomic status. In the setting of a facial fracture, the primary recommendations will likely come from a surgical team such as ENT or oral and maxillofacial surgery. They may elect to admit or transfer the patient for inpatient and operative management, as in the case of our Power Slap fighter. Some mandibular fractures may allow for discharge with very close follow-up. However, there should be strong consideration for admission or transfer for admission and operative management in cases of fractures deemed ‘open’, poor follow-up, and in pediatric patients [14].

## Conclusions

Power Slap fighting presents a novel and increasingly relevant mechanism of injury that emergency clinicians must be prepared to recognize and manage. Unlike traditional combat sports, this competition is built around repetitive, unprotected strikes to the lower face, with no opportunity for defensive movement. This unique format places participants at high risk for maxillofacial trauma, airway compromise, and long-term neurologic sequelae. The case described herein highlights a comminuted mandibular fracture requiring surgical intervention and revision, with persistent sensory deficits - an outcome that underscores the severity of injuries this sport can produce.

As the sport continues to gain traction, EDs - particularly those in military or rural settings - should be equipped with protocols for evaluating facial trauma, initiating timely surgical consultation, and ensuring appropriate imaging and follow-up. Clinicians should also be aware of the sport's minimal protective gear, the potential for delayed complications, and the psychosocial context in which these injuries may occur. While this patient was fortunate to receive prompt care, his case emphasizes the importance of early recognition, airway vigilance, and interdisciplinary coordination in the management of Power Slap-related injuries. Understanding this emerging injury pattern will be key to protecting the growing population of participants and ensuring that what may begin as a spectacle does not end in significant disability.
